# Curators of Med. Department of University of Buffalo

**Published:** 1852-09

**Authors:** 


*Curators of Med. Department of University of Buffalo.*—We are requested to state that the names of the curators of the Med. Dept. of the University of Buffalo, residing in this city, were inadvertently left out of the list of curators in the annual circular for 1852. The names of the gentlemen referred to, are as follows:—Drs. E. Wallis, Samuel Carey, S. F. Mixer, and J. B. Samo.

